# Association between Waste Management and HBV among Solid Municipal Waste Workers: A Systematic Review and Meta-Analysis of Observational Studies

**DOI:** 10.1155/2013/692083

**Published:** 2013-10-09

**Authors:** Carmela Romana Natalina Corrao, Angela Del Cimmuto, Carolina Marzuillo, Emanuele Paparo, Giuseppe La Torre

**Affiliations:** Department of Public Health and Infectious Diseases, Sapienza University of Rome, Piazzale Aldo Moro 5, 00185 Rome, Italy

## Abstract

*Aim*. To conduct a systematic review of this relationship using available published observational studies in the field of solid municipal waste treatment. *Methods*. The review of the scientific literature was based on Medline and Scopus databases up to December 2012, using the keywords HBV, waste, solid, treatment, workers, disposal, and refuse in different combinations. *Results*. 160 studies were found and checked. Finally, 5 observational studies were considered suitable, all cross-sectional. The pooled proportion of HBs-Ag considering all the studies was 11% (95% CI: 5–21%), and considering the high quality studies only, this proportion was 14% (95% CI: 6–24%). The pooled proportion of HBs-Ab positivity among waste workers considering all the studies was 14.2% (95% CI: 1.4–37.2%), and considering the high quality studies only, this proportion was 24% (95% CI: 18–30%). The pooled proportion of HBc-Ab positivity among waste workers considering all the studies was 24% (95% CI: 6–49%). The pooled estimation of the risk of HBV positivity (HBsAg) among exposed was OR = 2.39 (95% CI: 0.88–6.52). *Conclusion*. In conclusion, waste workers need to be vaccinated against HBV infection since they are at risk of acquiring this infection through the exposure to potentially infected waste.

## 1. Introduction

Hepatitis B (HBV) is considered an important hazard for the general and working population. The virus transmission occurs through blood, blood products, and other body fluids, such as semen, in the following cases: sexual contact, drug use with shared needles, transfusions with blood, or blood products and medical (including dental) practices where inadequate infection control precautions are employed [1].

HBV infection affects the liver, where it can cause acute and chronic diseases (liver infections, cirrhosis). Actually, chronic infections with hepatitis B virus (HBV) and hepatitis C virus (HCV) are also considered responsible of liver cancer and are classified by the International Agency for Research on Cancer (IARC) as carcinogenic to humans (group 1) [2].

According to WHO, each year two billion people worldwide are infected by HBV, among which 600,000 died and more than 240 million develop chronic liver diseases. Hepatitis B is endemic in China and other Asian countries. It is also estimated that 2–5% of the population in the Middle East and India and less than 1% in Western Europe and North America are chronically infected by HBV [2].

HBV is involved especially in occupational percutaneous exposures of health care workers (HCWs). The risk of infection is increased by accidents at work and for HBV it is estimated to be 4.29 times higher [3].

Worldwide, the annual fraction of health care workers exposed to bloodborne pathogens is estimated to be 5.9% for HBV and the HBV infections to about 66.000 [4].

On the other hand, the proportion of HBV infections attributable to percutaneous occupational exposure is estimated to be 40%–65% in developing regions and less than 10% in developed regions. According to WHO, in Europe occupational percutaneous exposures for HBV transmission in health care workers each year account for 304,000, whereas the probability of this infection after an occupational exposure should be between 18% and 37% [5, 6].

Hepatitis B is preventable with the available vaccine, which is considered safe and effective. The hepatitis B vaccine has been available since 1982 and is considered effective in preventing 95% of infections and HBV-related chronic diseases, including liver cancer. The immunization of workers reduces their susceptibility to infection and infections' number. Long-term immunity offered by vaccines against HBV was recently reported in 50% of health workers 30 years after vaccination [7].

Other workers involved by contact with blood and body fluids are considered potentially exposed to HBV (barbers, military personnel, public safety workers, fire fighters, butchers, embalmers, and sewage treatment). The risk of HBV infection is also related to recent activity in the field of cosmetics (tattooing and piercing), and even the international travelers (missionaries and long-term aid workers) are considered at increased risk because of hygienic conditions in the destination countries [8].

Municipal solid waste workers (MSWW) are also considered at risk of contracting HBV infection, and the first reports date back to 1975 [14].

However, little is known concerning the relationship between HBV infection and nonbiomedical occupational exposure. So, the aim of the present study is to conduct a systematic review of this relationship using available published observational studies in the field of solid municipal waste treatment.

## 2. Methods

### 2.1. Identification of Relevant Studies

The review of the literature was based on Electronic medical databases. The search was applied to Medline and Scopus databases. For each database, we used the keywords “HBV and waste,” “HBV and solid waste,” “HBV and solid waste treatment,” “HBV and waste workers,” “HBV and solid waste workers,” “HBV and solid waste disposal workers,” and “HBV and refuse workers.”

Articles were selected from the medical area of PubMed and Scopus. In addition, all potentially relevant studies found in the references of the selected articles were included. Only articles published until December 2012 were considered.

### 2.2. Data Extraction and Quality Assessment

**Figure 1:**
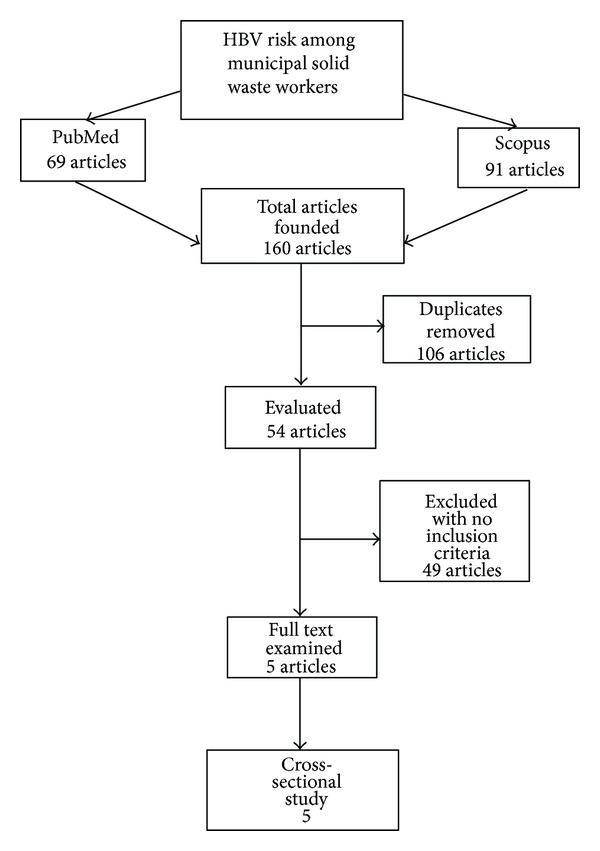
Flow chart of the selection of the studies.

The selection of articles, performed according to the PRISMA statement [15], is shown in the flowchart (Figure 1).

The duplicate articles emerging by PubMed and Scopus consultation were removed. 

The inclusion criteria were as follows:

- observational studies;
- English language;
- availability of full text;
- data concerning HBV markers (at least HBs-Ag, HBc-Ab, and HBs-Ab).

The full texts of included publications were analyzed by two different researchers independently. 

Quality assessment for all included studies was performed using a quality scoring for observational studies assessment [16].

Any disagreement between the two researchers was solved with a discussion and a second examination.

### 2.3. Statistical Analysis

Two different analyses were carried out. The first one included all selected observational studies that evaluated the prevalence of HBV infection in these workers; the second one included only the association between the main exposure considered and the HBV infection. This association was assessed using the odds ratio (OR) measure with a relative 95% confidence interval (95% CI). The Chi-square test was computed to evaluate studies' heterogeneity, using the random effect model when the test highlighted differences between studies and the fixed effect model when no significant differences were shown [17]. The level of significance was set at *P* < 0.05.

The sensitivity analysis was conducted including only studies with a quality score >10 (good quality studies).

Meta-analysis was performed using StatsDirect software.

## 3. Results

### 3.1. Identification of Relevant Studies

A total of 160 studies were found through PubMed and Scopus databases: 69 and 91 articles were retrieved, respectively. Of these, 106 articles were excluded because of duplicates of Medline and Scopus outcomes, whereas 49 were excluded because they did not fit with the inclusion criteria.

Finally, 5 observational studies were considered suitable, all cross-sectional, whose characteristics are presented in Table 1 [9–13].

### 3.2. Quality Assessment, Data Extraction, and Statistical Analysis

**Figure 2:**
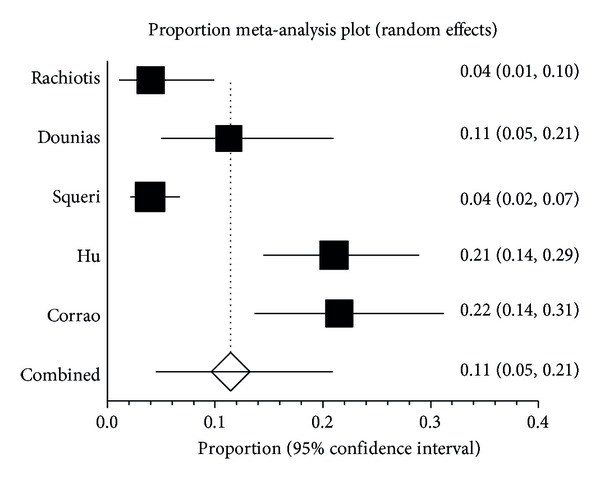
Proportion meta-analysis plot of HBs-Ag among exposed.

Four out of five studies were considered of high quality (see Table 1), with a prevalence of HBs-Ag among exposed ranging from 3.98% to 21.5%. The pooled proportion considering all the studies was 11% (95% CI: 5–21%, random effect model) (Figure 2).

Considering high quality studies only, this proportion was 14% (95% CI: 6–24%, random effect model) (data not shown).

**Figure 3:**
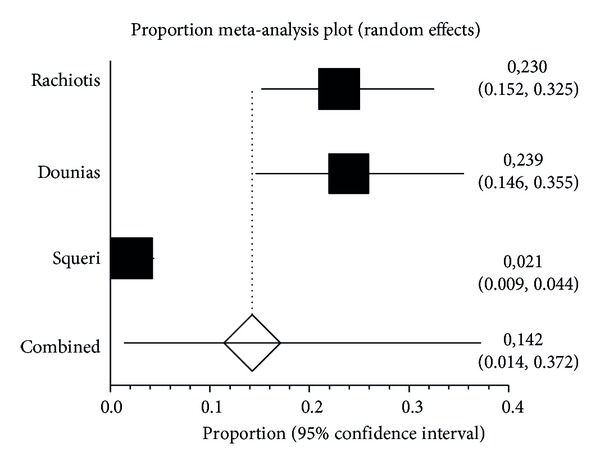
Proportion meta-analysis plot of HBs-Ab among exposed.

The pooled proportion of HBs-Ab considering all the studies was 14.2% (95% CI: 1.4–37.2%, random effect model) (Figure 3).

Considering high quality studies only, this proportion was 24% (95% CI: 18–30%, random effect model) (data not shown).

The pooled proportion of HBc-Ab considering all the studies was 24% (95% CI: 6–49%, random effect model) (data not shown).

Only two studies calculated or gave the results for the exposed (being the exposure, the occupational exposure to waste) and the nonexposed, giving the results as ORs. The pooled estimation of the risk of HBV positivity (HBsAg) among exposed was OR = 2.39 (95% CI: 0.88–6.52) (fixed effect model: *P* for homogeneity = 0.912) (data not shown).

## 4. Discussion

HBV infection is particularly high in some population groups, such as homeless and immigrants, and according to WHO forecast, it will be the third cause of death for infectious diseases in 2030 among industrialized countries [18].

This paper systematically reviewed the association between professional exposure to waste and HBV infections among workers. For each selected study, it was possible to calculate the prevalence of HBs-Ag, while the calculation of the prevalence of HBs-Ab was possible only by using 3 out of 5, and the prevalence of HBc-Ab only by using 2 out of 5 (Table 1).

The work of Corrao et al., published in 1985, treats infections caused by hepatitis A and hepatitis B in the refuse workers of Asti (Turin, Italy) by analyzing a sample of a total of 93 workers. The prevalence of HBs-Ag of exposed workers is 21.5% (20/93) [13].

The prevalence of HBs-Ag derived from the study by Hu et al., published in 2003, employees of municipal solid waste incinerators in Taiwan, was found to be 21.1% (28 cases out of 133 workers considered) [12].

The work published in 2005 by Dounias et al. shows the values of the markers of hepatitis B virus (HBs-Ag, HBs-Ab, and HBc-Ab) detected in municipal solid waste workers in Keratsini Greece. Out of a total of 159 municipal solid waste workers analyzed 71 were determined to be exposed. The prevalence of HBs-Ag exposed is 23.9% (17/71) and that of HBs-Ab is 11.2% (8/71), while that of HBc-Ab was 12.7% (9/71). In the study, cases of hepatitis B among nonexposed were shown, so, it was possible to calculate the OR (OR = 2.49, 95% CI: 0.97–8.57) to produce a risk estimate [11].

Also, in the work of Squeri et al. the presence of serological markers of hepatitis B virus (HBs-Ag, HBs-Ab, and HBc-Ab) between 327 municipal solid waste workers of Messina was considered. The prevalence calculated for each marker was found to be equal to 3.98% for HBs-Ag (13/327), 2.1% for HBs-Ab (7/327), and 35.8% for HBc-Ab (117/327) [10].

Another study selected where it was possible to calculate the OR is the most recent, published in 2011, of Rachiotis et al. where the results of a survey were carried out in Greece on 100 waste collectors with a prevalence of 4.0% for HBs-Ag and a prevalence of 23.0% for HBs-Ab (23/100). The estimated risk was 2.21 (95% CI 0 : 39 to 12 : 33) [9].

We need to take into account that microbial air contamination is common at controlled landfills [19] or at waste selection plants [20]. A study carried out by Boccia et al. [21], concerning the health monitoring of solid waste management workers, underlines the need of implementing specific risk prevention training, such as the use of individual protection devices.

Our study underlines the need for this kind of workers to be vaccinated against HBV infection. This vaccine has been available since the beginning of the 1980s, and in some countries, such as Italy, it is free of charge for workers employed in the different processes of waste management [22], as well as for biomedical workers [23]. 

The more recent Directive 2000/54/CE of the European Parliament and of the Council of September 18, 2000, (concerning the protection of workers towards biological risk at work) strengthens workers' health and safety in relation to biological agents, paying particular attention to determination and assessment of risks, employers' obligations (*replacement, reduction of risks, information for the competent authority, hygiene and individual protection, information and training of workers, list of exposed workers, consultation and participation of workers, notification to the competent authority*), and health surveillance. Regarding health surveillance, the Directive establishes that the risk assessment “*should identify those workers for whom special protective measures may be required” *and* “when necessary, effective vaccines should be made available for those workers who are not already immune to the biological agent to which they are exposed or are likely to be exposed*.” Also, to Annex III (community classification) it is specified that HBV effective vaccine is available [24].

Even if this is the first systematic review and meta-analysis of this issue, we must acknowledge some limitation. It deals mainly with the study-design type of the observational studies retrieved (cross-sectional). Moreover, we have to recognize that sometimes the lack of data from the single study did not make possible more robust estimations of the pooled prevalences and ORs. 

In conclusion, waste workers should be vaccinated against HBV infection since they are at risk of acquiring this infection through the exposure to potentially infected waste. 

## Figures and Tables

**Table 1:** Characteristics of the selected studies.

Authors	Year of publication	Number of participants (exposed)	Prevalence of HBs-Ag among exposed	Prevalence of HBs-Ab among exposed	Prevalence of HBc-Ab among exposed	OR	Quality score
Rachiotis et al. [9]	2012	208 (100)	4% (4/100)	23% (23/100)	n.a.*	2.21 (0.39–12.33)	18
Squeri et al. [10]	2006	327 (327)	3.98% (13/327)	2.1% (7/327)	35.8% (117/327)	n.a.*	8
Dounias et al. [11]	2005	159 (71)	11.2% (8/71)	23.9% (17/71)	12.7% (9/71)	2.49 (0.71–8.57)	17
Hu et al. [12]	2003	133 (133)	21.1% (28/133)	—	—	n.a.*	17
Corrao et al. [13]	1985	93 (93)	21.5% (20/93)	—	—	n.a.*	11

*n.a.: not applicable, since the population was made by exposed people only.
